# Barriers to apply cardiovascular prediction rules in primary care: a postal survey

**DOI:** 10.1186/1471-2296-8-1

**Published:** 2007-01-03

**Authors:** Klaus Eichler, Marco Zoller, Peter Tschudi, Johann Steurer

**Affiliations:** 1 Horten Centre for patient oriented research, University hospital of Zurich, CH-8091 Zurich, Switzerland; 2 Zürcher Ärztegemeinschaft zmed, Grütlistrasse 36, CH-8002 Zurich, Switzerland; 3 Institut für Hausarztmedizin, University of Basle, Bläsiring 160, CH-4057 Basle, Switzerland

## Abstract

**Background:**

Although cardiovascular prediction rules are recommended by guidelines to evaluate global cardiovascular risk for primary prevention, they are rarely used in primary care. Little is known about barriers for application. The objective of this study was to evaluate barriers impeding the application of cardiovascular prediction rules in primary prevention.

**Methods:**

We performed a postal survey among general physicians in two Swiss Cantons by a purpose designed questionnaire.

**Results:**

356 of 772 dispatched questionnaires were returned (response rate 49.3%). About three quarters (74%) of general physicians rarely or never use cardiovascular prediction rules. Most often stated barriers to apply prediction rules among rarely- or never-users are doubts concerning over-simplification of risk assessment using these instruments (58%) and potential risk of (medical) over-treatment (54%). 57% report that the numerical information resulting from prediction rules is often not helpful for decision-making in practice.

**Conclusion:**

If regular application of cardiovascular prediction rules in primary care is in demand additional interventions are needed to increase acceptance of these tools for patient management among general physicians.

## Background

General physicians play an important role for individual directed primary prevention of cardiovascular disease during patient consultation. Their recommendations on life style changes or additional management decisions (like drug treatment) should be based on estimates of global absolute risk [1-3].

Estimation of global cardiovascular risk is challenging and cardiovascular prediction rules have been developed as useful tools for risk estimation [1-3]. Studies have shown, however, that general physicians rarely apply cardiovascular prediction rules [4,5]. Little is known about barriers for application in daily practice [6,7].

We performed a postal survey among general physicians in two Swiss Cantons to evaluate barriers impeding the application of cardiovascular prediction rules for primary prevention.

## Methods

### Study design and participants

We conducted a postal survey among physicians in the field of general Medicine (general practitioners and specialists for general internal medicine) working in their own practice in two Swiss regions (Canton of Zurich and Canton of Aargau). The address databases were provided by the regional physician organisations, which cover all practising physicians in each region (Zürcher Ärztegesellschaft for the Canton of Zurich and Aargauischer Ärzteverband for the Canton of Aargau).

In a first survey the numbered questionnaires were sent to all eligible physicians in the Canton of Aargau (n = 386) and to a randomly selected sample in the Canton of Zurich (n = 386 out of 1337). We provided a short introduction letter with the same mailing to explain the context of the study. After three weeks we conducted a second mailing (reminder) among those physicians who did not respond to our first mailing.

The survey was conducted quasi-anonymously to avoid bias caused by social desirability. To reduce the logistic effort for the second mailing, we traced each participant using a tracking number. The Institute for Social and Preventive Medicine, University of Zurich, guaranteed blinding (the authors of the study could not link answers with names of participants).

### Questionnaire development

Based on a literature search and the results of a recent workshop with experts from the health care system including general physicians [8] we first gathered potential obstacles for application of cardiovascular prediction rule in daily routine.

In a next step, we discussed these possible barriers in a focus group of general physicians in order to derive a set of them, which may explain why cardiovascular prediction rules are not or only rarely applied in practice. Twenty potential obstacles were listed and pilot tested for comprehensibility and additional items in a group of 8 general physicians, who were not involved in the questionnaire development. We interviewed the participating physicians about their understanding and perceptions of the content of the questionnaire. Unclear wording was amended accordingly and integrated in the final form of the questionnaire.

Finally, the questionnaire [see Additional file 1] contained a set of potential barriers covering three different dimensions: (1) Lack of knowledge (like "no overview about all the existing prediction rules"), (2) distrust (like "distrust in the validity of instruments", or "not all important risk factors are included in the prediction rules"), and (3) practicability aspects (like "risk estimation is time consuming"). Labelling of dimensions was not visible for participating physicians. One open-ended question asked for other reasons for "rarely"- or "never"-use of prediction rules.

In addition, we collected data about frequency of use of prediction rules in three categories ("often" defined as at least once a week; "rarely" defined as less than once a week; and "never"). We also asked general physicians to rate usefulness of support measures (like "journal articles" or "workshops") to resolve open questions concerning prediction rules.

### Outcome

Only physicians answering that they "never" or "rarely" use prediction rules were requested to answer questions about obstacles. Our primary interest pertained the frequency of specific obstacles for application of cardiovascular prediction rules among physicians who reported to "rarely" or "never" use those rules.

### Analysis

For our descriptive analysis we used medians and inter-quartile ranges (IQR: 25% and 75% percentiles) for continuous data and proportions for categorical data.

Data analysis was performed with SPSS for Windows, version 12.0.1 (SPSS Inc., Chicago, Illinois).

## Results

**Figure 1:**
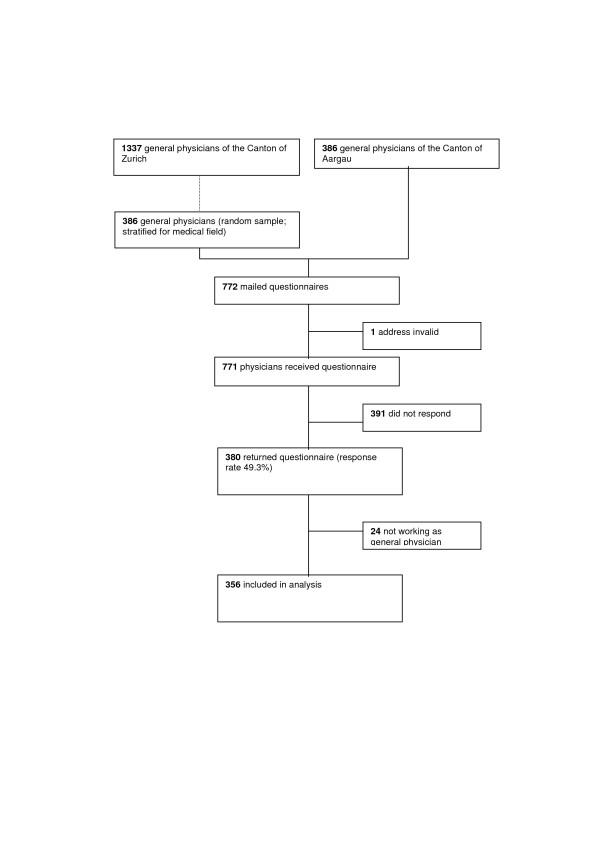
Study flow.

Out of all 1723 general physicians registered in the two Swiss regions we recruited a study sample of 772 doctors (study flow figure 1). The response rate was 49.3% (380 of 771; one address was invalid). For our further analysis we excluded 24 participants who stated in the questionnaire that they did not work as general physicians anymore. Thus our final dataset consisted of 356 participants. Data quality was high (range for completeness of data for the primary interest: 91 – 96 %).

For characteristics of participants see table 1. The sample consisted of experienced doctors (median time interval since Medical license 25 years, IQR: 18 – 29), most of them working as general practitioners (63.7%).

**Table 1:** Characteristics of participants and use of cardiovascular prediction rules

**Age**	**n (%) **(if not stated otherwise)
Median (IQR)	51 (45 – 57) years
**Sex**	
Male	276 (78.9%)
**Medical specialties of general physicians**	
General Practitioners	227 (63.7%)
Specialists for General Internal Medicine	102 (28.7%)
Additional medical specialties covered by general physicians (like Gastroenterology, Gynaecology, or Diabetology)	43 (12.1%)
**Years since Medical license**	
Median (IQR)	25 (18 – 29) years
**Use of cardiovascular prediction rules**	
"often" (at least once a week)	80 (22.5%)
"rarely" (less than once a week)	156 (43.8%)
"never"	107 (30.1%)

356 participants included. Some totals may not be 100% due to missing data or multiple answers

About three quarters of general physicians (73.9%; 263/356) reported that they "rarely" or "never" used cardiovascular prediction rules, while 22.5% (80/356) reported to use them "often" (13 respondents with missing data). The various prediction rules, which are applied in our sample of general physicians, are listed in table 2.

**Table 2:** Applied cardiovascular prediction rules

**Prediction rule**	**Outcome**	**n (%)**
PROCAM-score	Coronary death, myocardial infarction	24 (30%)
EU-SCORE	Cardiovascular death	14 (18%)
Framingham score	Coronary death, myocardial infarction, angina pectoris	11 (14%)
AGLA-score (PROCAM-derived Swiss risk score)	Coronary death, myocardial infarction	33 (39%)
Others (like New Zealand Guidelines)	Coronary death, myocardial infarction, angina pectoris	4 (5%)

80 participants included (using prediction rules "often"). Total is not 100% due to multiple answers

### Reasons not to apply cardiovascular prediction rules

**Figure 2:**
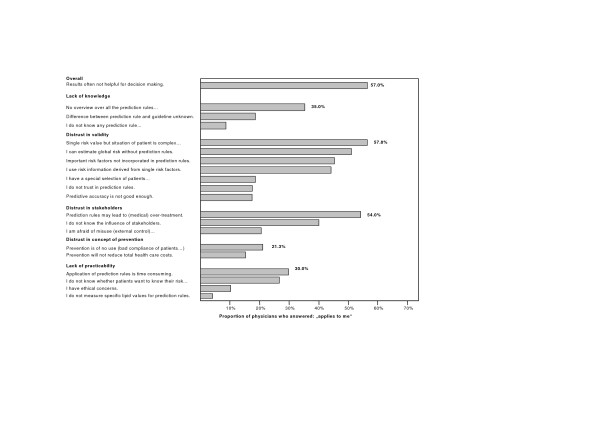
**Barriers for application of cardiovascular prediction rules**. 263 participants (using prediction rules "rarely" or "never").

Among the 263 doctors, who used cardiovascular prediction rules "rarely" or "never", the most often stated barriers to apply these instruments were doubts concerning over-simplification of risk assessment and potential risk of over-treatment (for detailed results see figure 2). 58% (152/263) stated that they did not use prediction rules because "a single risk value does not take into account the complex situation of the patient" and 54% (142/263) because "the results of prediction rules may lead to over-treatment (over-use of statins)". 133 of 263 physicians (51%) agreed to the statement "I do not use prediction rules as I know my patients well and can estimate their global risk correctly without a prediction rule".

Statements in the dimension "lack of knowledge" or "aspects of practicability" were chosen much less frequently (range: 4% – 35%). Overall, for 57% (150/263) of respondents "the results of prediction rules often are not helpful for decision making in practice". The open-ended question for other reasons did not reveal different information compared to the predefined categories.

### Support to resolve open questions concerning prediction rules

More than one quarter of all respondents (26.7%) stated that they did not need additional support to resolve open questions concerning cardiovascular prediction rules (table 3). Additional information about prediction rules was rated helpful in form of journal articles (26.1%), lectures (19.9%), workshops (27.5%), and more simple prediction rules (24.2%).

**Table 3:** Support suggested as helpful to resolve open question about cardiovascular prediction rules.

**Support**	**Proportion of respondents (n)**
No additional support desired	26.7% (95/356)
Workshops	27.5% (98/356)
Journal articles	26.1% (93/356)
More simple prediction rules	24.2% (86/356)
Lectures	19.9% (71/356)

## Discussion

About three quarters of general physicians rarely or never use cardiovascular prediction rules in their daily work. Most often stated barriers to apply them are doubts concerning over-simplification of risk assessment with these instruments and potential risk of over-treatment. More than half of respondents stated that they were able to estimate cardiovascular risk correctly without such rules and that information generated with prediction rules is often not helpful for decision-making.

### Validity of the findings

Our questionnaire was not formally tested for internal consistency and validity. However, we judge the face validity of our instrument as adequate. We choose a thorough, stepwise development process (literature search; interactive workshop; focus group discussion; pilot testing) that included experts from the health care system and experienced general practitioners. The response rate of about 50% and the completeness of data were acceptably high for a postal survey. In addition, we believe that the quasi-anonymous data collection has further improved validity of results. However, we could not thoroughly evaluate if non-responders of our survey were comparable to responders due to protection of data privacy. The true application rate of cardiovascular prediction rules in daily routine may even be lower as motivated physicians may be over-represented in our study.

Our data fit well with recent qualitative research identifying obstacles to apply cardiovascular risk tables in routine general practice [7,9]. In those studies barriers related to the instrument (like distrust in the validity of prediction rules), to aspects of environment and society (like marketing efforts of pharmaceutical industry) and to practice routine (like management of single risk factors) were some of the relevant factors. These obstacles were among the most frequent reasons for non-application in our study. Scepticism in cardiovascular prediction rules seems to be considerable among our respondents. The rate of "rarely"- or "never"-users was 74%, which is beyond the results of about 50% reported in recent surveys [4,5] but fits well with the results of a postal survey among Norwegian general practitioners [10]. In addition, the application of more than five prediction rules with partly diverse outcomes may reflect a general uncertainty even among "often-users" which prediction rule to apply for daily practice.

### Estimation of cardiovascular risk

Argumentation of general physicians is not free of contradiction. While 58% argue that a single risk value derived from prediction rules is an oversimplification, 46% state that they use information derived from single risk factors for their decisions. Application of prediction rules may be regarded as oversimplification but evidence exists that even experienced family doctors may estimate global baseline risk less precise than prediction rules despite the moderate accuracy of these tools [11-13]. Risk estimation without prediction rules may be specifically difficult if several risk factors are only mildly or moderately elevated. Family doctors probably misinterpret comprehensive knowledge about their patients as an ability to correctly estimate their global cardiovascular risk. In addition, among some doctors a "treatment reflex" with prescription of lipid-lowering drugs for persons with isolated hyperlipidemia may exist (irrespective of a possibly low global risk like in persons without hypertension, without smoking, and of young age) [4]. The data from our survey support this hypothesis. Thus persons with low absolute risk may receive medical treatment, which is in conflict with the frequently stated concern about medical over-treatment.

### Implications for practice

A possible explanation for the rejection of prediction rules might be the judgement that statistical risk information seems to be incompatible with comprehensive and individualised patient care. Acceptance of prediction rules might be higher if family doctors understand risk information as an additional tool beside their clinical experience to tailor recommendations to the individual patient. Given suitable communication measures (like communication training for physicians; educational material for patients; or graphical presentations of individual risk values with average risk estimates of the same age group) [14], cardiovascular risk estimates derived from prediction rules may strengthen motivation for life style changes as the first choice intervention or drug treatment, where necessary [1,3].

Controlled trials evaluating the impact of prediction rules on patient management have concentrated on technical aspects (like computerized calculation of global risk and standardized documentation) [15,16]. However, little is known about which educational intervention is most effective to overcome distrust in prediction rules. A recent Cochrane review has favored interactive workshops rather than didactic presentations to influence professional practice [17]. Respondents of our survey do not seem to favor a specific form of medical education, if any, to resolve their open questions concerning prediction rules. Specific tailored interventions to overcome barriers (like educational outreach visits with audit and feedback as well as computerised reminders linked to the medical record system) have been evaluated in a recent controlled trial [18]. The intervention had no or little impact on the frequency of formal risk assessment.

## Conclusion

If regular application of cardiovascular prediction rules in primary care is in demand, additional interventions are needed to increase acceptance of these tools for patient management among general physicians.

## Competing interests

The author(s) declare that they have no competing interests.

## Authors' contributions

JS had the study idea. KE, MZ, PT and JS designed the study. KE was responsible for data acquisition and analysis. All authors made substantial contributions to interpretation of the data. KE and JS drafted the manuscript. All authors were involved in revising the manuscript critically for important intellectual content and have given final approval of the version to be published.

## Pre-publication history

The pre-publication history for this paper can be accessed here:



## Supplementary Material

Additional file 1 **Questionnaire: Potential barriers for application of cardiovascular prediction rules**. For descriptive purposes questions are sorted according to dimensions. Labelling of dimensions was not visible for participating physicians. Click here for file
