# Supplementary material for: Barriers to apply cardiovascular prediction rules in primary care: a postal survey
Source: BMC Fam Pract. 2007 Jan 3;8:1. doi: 10.1186/1471-2296-8-1 (PMC1766351; doi:10.1186/1471-2296-8-1)
Supplement: Additional file 1 — Questionnaire: Potential barriers for application of cardiovascular prediction rules. For descriptive purposes questions are sorted according to dimensions. Labelling of dimensions was not visible for participating physicians. [file 1471-2296-8-1-S1.pdf]

Additional file 1:

Questionnaire: Potential barriers for application of cardiovascular prediction rules. For descriptive purposes questions are sorted according to dimensions. Labelling of dimensions was not visible for participating physicians.

**Why I "rarely" or "never" apply prediction rules:**
**Overall:**<br><br>The results of prediction rules are often not helpful for decision making in practice
**1) Domain "Lack of knowledge":**
I cannot keep track of all existing prediction rules and do not know which one to choose.
I do not know the difference between prediction rules and guidelines.
I do not know any prediction rule.
**2.1) Domain "Distrust in the validity of instruments":**
A single risk value does not take into account the complex situation of the patient.
I know my patients thoroughly and can estimate their global risk correctly without a prediction rule.
Prediction rules do not contain risk factors, which are important in my opinion (like occupational factors, stress, or obesity).
I use information from single risk factors for decision-making (like hypertension or hyperlipidemia); thus I know how to treat….

Additional file 1: **Potential barriers for application of cardiovascular prediction rules** (continued):

I cannot apply prediction rules due to the special patient selection (like ethnic minorities, or groups with social deprivation).
I do not trust prediction rules (I remember persons with a low risk who experienced a myocardial infarction…).
The predictive accuracy of prediction rules is not high enough.
**2.2) Domain “Distrust in stakeholders”:**
The results of prediction rules may lead to over-treatment (like over-use of statins).
I do not know which stakeholders or interest groups were engaged in the development of prediction rules.
I am afraid of misuse of prediction rules (like external control by health insurances or health authorities).
**2.3) Domain “Distrust in the concept of prevention”:**
Prevention is of no use (if someone does not change life style anyway, he will not do this with “risk information”; bad compliance).
Even if prevention may be useful for single persons, total costs of the health care system will not be reduced.
**3) Domain “Aspects of practicability”:**
Application of prediction rules and patient counselling is time consuming.
I do not know if patients want to know their individual risk at all.
I have ethical concerns.
For risk estimation specific lipid values are necessary, which I do not determine in my office.

Additional file 1: **Potential barriers for application of cardiovascular prediction rules** (continued):

**4) Open ended question:**
Are there other reasons why you never or rarely use prediction rules? If yes, please write these reasons down in the space below.
